# Impact of Compliance with a Care Bundle on Acute Kidney Injury Outcomes: A Prospective Observational Study

**DOI:** 10.1371/journal.pone.0132279

**Published:** 2015-07-10

**Authors:** Nitin V. Kolhe, David Staples, Timothy Reilly, Daniel Merrison, Christopher W. Mcintyre, Richard J. Fluck, Nicholas M. Selby, Maarten W. Taal

**Affiliations:** 1 Department of Renal Medicine, Royal Derby Hospital, Uttoxeter Road, Derby, DE22 3NE, United Kingdom; 2 Department of Acute Medicine, Royal Derby Hospital, Uttoxeter Road, Derby, DE22 3NE, United Kingdom; 3 Department of Information Management and Technology, Uttoxeter Road, Derby, DE22 3NE, United Kingdom; 4 Division of Medical Sciences and Graduate Entry Medicine, University of Nottingham, Uttoxeter Road, Derby, DE22 3NE, United Kingdom; University of Florida, UNITED STATES

## Abstract

**Background:**

A recent report has highlighted suboptimal standards of care for acute kidney injury (AKI) patients in England. The objective of this study was to ascertain if improvement in basic standard of care by implementing a care bundle (CB) with interruptive alert improved outcomes in patients with AKI.

**Methods:**

An AKI CB linked to electronic recognition of AKI, coupled with an interruptive alert, was introduced to improve basic care delivered to patients with AKI. Outcomes were compared in patients who had the CB completed within 24 hours (early CB group) versus those who didn’t have the CB completed or had it completed after 24 hours.

**Results:**

In the 11-month period, 2297 patients had 2500 AKI episodes, with 1209 and 1291 episodes occurring before and after implementation of the AKI CB with interruptive alert, respectively. The CB was completed within 24 hours in 306 (12.2%) of AKI episodes. In-hospital case-fatality was significantly lower in the early CB group (18% versus 23.1%, p 0.046). Progression to higher AKI stages was lower in the early CB group (3.9% vs. 8.1%, p 0.01). In multivariate analysis, patients in the early CB group had lower odds of death at discharge (0.641; 95% CI 0.46, 0.891), 30 days (0.707; 95% CI 0.527, 0.950), 60 days (0.704; 95% CI 0.526, 0.941) and after a median of 134 days (0.771; 95% CI 0.62, 0.958).

**Conclusions:**

Compliance with AKI CB was associated with a decrease in case-fatality and reduced progression to higher AKI stage. Further interventions are required to improve utilization of the CB.

## Introduction

There is growing evidence that the incidence of acute kidney injury (AKI) is increasing, because of increasing age and comorbidities of hospitalized patients and increasing prevalence of risk factors for AKI, especially chronic kidney disease [1–3]. In addition, AKI is associated with increasing length of stay and high mortality rates. A recent National Confidential Enquiry in Patient Outcome and Death (NCEPOD) study highlighted poor standards of basic medical care in a significant proportion of hospitalised patients with AKI in England[4]. In patients with AKI requiring dialysis, various strategies to decrease mortality with innovative treatments like biocompatible dialysers, early initiation of dialysis and higher dialysis dose have not shown to be effective[5, 6]. In non-dialysis requiring AKI, interventions like dopamine, renal vasodilators, diuretics, growth factors and recently erythropoietin have all been proved to be futile and in some cases deleterious [7–9]. It is extremely important therefore, to focus on improving and standardizing basic care to ensure that all patients receive the best possible management. It is quite clear from recent studies that the majority of patients with AKI are cared for by non-nephrologists [10]. Clinical diagnosis of AKI has until now, relied on clinicians having high index of suspicion on the basis of clinical history and examination. Quite often, blood investigations are delayed, or if done, a small rise in creatinine is ignored. Added to this is the challenge of monitoring urine output, which is quite often not accurately measured. From a workload perspective it may not be feasible for a nephrologist to see every patient with AKI and improving basic care may go a long way to improving clinical outcomes. Various national organizations have produced guidelines emphasizing the importance of basic standards of care[11, 12]. Recently, many organisations have produced educational packages and mobile applications with a view to improve clinical outcomes[13]. However, there have been no studies to assess clinical outcomes after implementation of an innovation to improve basic clinical care.

In our institute, we developed a single page AKI CB (CB), which consists of diagnostic and therapeutic interventions, designed to standardize and improve initial management and help non-nephrologists to intervene quickly to treat and prevent progression of AKI, thereby reducing its associated morbidity and mortality.

The aim of this study was to ascertain if the use of AKI CB was associated with improved patient outcomes as assessed by in-hospital mortality, AKI stage progression and length of stay.

## Materials and Methods

This single centre study was conducted at the Royal Derby Hospital, a 1139-bed tertiary care centre. Data were collected from February 2013 to December 2013 on all adult patients over the age of 18 years. An electronic recognition system for AKI has been in use since 2010 that allows prospective data collection for all cases of AKI [10]. The electronic recognition system was modified in December 2012, to incorporate the KDIGO definitions for AKI. In an effort to improve the clinical outcomes, intranet guidelines were introduced along with the electronic recognition and alerting system. In December 2012, a one-page paper version (S1 Fig) of the AKI CB was devised, which was subsequently incorporated into the hospital electronic patient record on 1^st^ February 2013 (S2 Fig). During this period, education on the AKI CB was given to all staff in acute areas of the hospital and a poster explaining the AKI CB was displayed in all clinical areas. The hospital already had electronic ordering of investigations and prescribing of medications in place. The CB consists of simple standardized investigations and interventions, reminding clinicians of the importance of thorough *Assessment*, *Urinalysis*, establishing a *Diagnosis*, planning *Investigations* and *Treatment* and at the same time issuing guidance about *Seeking* advice from nephrologist while caring for patients with AKI (AUDITS). All the steps in the CB are objective and the clinicians are requested to click yes or no for each step of the CB. On 1st August 2013, the AKI CB was coupled with an interruptive electronic alert, which was triggered by the first attempt to order blood tests or medications on patients who had been identified as having AKI (S3 Fig). The interruptive alert would warn the clinician about AKI and request them to complete the AKI CB. Once the AKI CB was completed, the clinician was able to request blood tests or medication. The clinician would be able to override the alert only after stating the reason. The location of patients was categorized into the following divisions—Medical Assessment Unit, Medical wards, Surgical & Orthopedic wards, Haematology and Oncology wards, Intensive Care and Surgical Step Down (high dependency unit) and Other wards.

A weekly report is automatically generated which captures patient location in the hospital, age, date of admission, date of discharge, finished consultant episode and survival status at the reporting date. For this study, we analyzed all AKI episodes, which had the AKI CB completed early (defined as within 24 hours of availability of the blood results) with those who either had AKI CB completed late (defined as after 24 hours of availability of the blood results) or not completed at all. The AKI patients who either had AKI CB completed late or not completed will be considered as not having AKI CB completed for this analysis. Community acquired AKI was defined as AKI within 24 hours of admission. Survival status of all patients was obtained in April 2014. The primary objective of this study was to assess the impact of use of AKI CB on patient safety markers, namely in-hospital case fatality, length of stay and progression of AKI stages. The designs of the paper and electronic CB along with the interruptive alert are provided in the supplementary appendix.

### Ethical approval

The study was discussed with research and development department of Derby Hospital NHS Foundation Trust and the National Research Ethics Service Committee, East of England—Cambridge Central. The study was deemed exempt from ethical approval because it involved audit of improvement in patient care following introduction of a CB to standardize care and met the criteria of service evaluation. Patients were not randomized to treatment groups and patient identifiable data was not used. All authors complied with the principles of Declaration of Helsinki. This is a retrospective study of service evaluation so it is possible that some of the authors may have interacted with a small number of the participants as part of their clinical care, but there was no interaction specific to this study.

### Statistical analysis

All analyses were performed using SPSS software (version 21). Continuous data were evaluated for normal distribution and are presented as the mean (95% confidence interval) or the median (interquartile range) as appropriate, and nominal data are presented as percentages. Comparison between the two groups was performed using chi-square test for nominal variables and t test for numerical variables where appropriate. Binary logistic regression was used to adjust known confounders and to test significant univariate associations with in-hospital mortality. Results are presented as odds ratios (OR) and 95% confidence intervals (CI). Data were analysed on each episode of AKI and included patients who had experienced multiple episodes of AKI in separate admission periods. We also performed cox regression analysis adjusting for age, gender, ethnicity, type of admission, maximum AKI stage reached in the admission, Charlson’s comorbidity score and CB completion within 24 hours, to assess if the survival advantage at discharge persisted at follow up. For cox proportional hazard analysis, we performed log minus log plot for categorical variables to confirm that there was no violation of the proportional hazards assumption. For numerical variables we plotted partial residuals against time to confirm there was no trend. All tests were two-tailed, and p<0.05 was considered significant.

## Results

**Fig 1:**
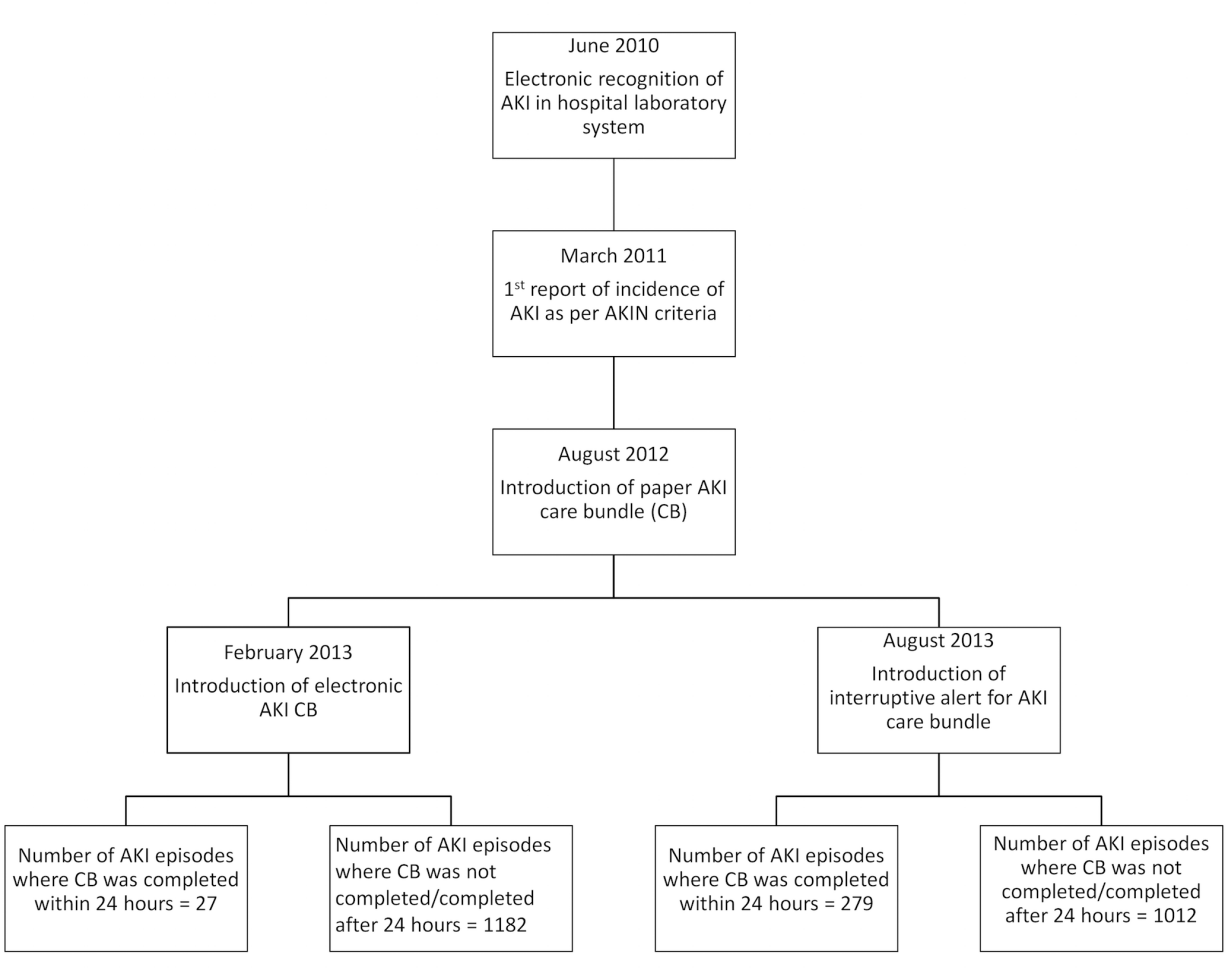
Study flow chart.

A total of 2500 episodes of AKI occurred in 2297 patients over the 11-month period, with 1209 and 1291 episodes occurring before and after the implementation of the AKI CB and interruptive alerts, respectively (Fig 1). 203 patients had separate admissions for repeat episodes of AKI. Overall, AKI CB completion rate was 12.2%. The AKI CB was completed within 24 hours in 2.2% (27) episodes of AKI prior to the interruptive alert and in 21.6% (279) episodes after the interruptive alert, p<0.001. There was delay of more than 24 hours to complete the CB in 0.2% (10) episodes before the interruptive alert and in 9.2% (119) episodes after the interruptive alert. CB was not completed in rest of the AKI episodes.

Table 1 shows baseline demographics of patients with AKI according to the timing of CB completion. Overall, patients who developed AKI were elderly with mean age of 76.6 (95% CI 76.1, 77.2) years and 91.4% were of white ethnicity. The median time taken to complete the AKI CB was 5 hours and 39 minutes (IQR 1 hour 57 minutes, 27 hours 16 minutes) from the availability of blood test result. The overall in-hospital case fatality was 22.4%. The median follow-up was 134 days (interquartile range 35 to 267 days). Age, ethnicity and Charlson’s comorbidity score did not differ between the two groups. Patients who had CB completed early tended to have higher AKI stage and a higher proportion had emergency admissions. There were 519 patients with stage 3 AKI, of which, only 15.7% had care bundle completed within 24 hours in contrast to 84.3% of stage 3 AKI who did not have care bundle completed (Table 2). In stage 3 AKI, there was no statistical difference in care bundle completion between community and hospital acquired AKI.

**Table 1:** Characteristics of patients according to Care Bundle completion.

		Care Bundle completion n (%)			p value
		Within 24 hours	Not completed or completed after 24 hours	Total	
Number of AKI episodes		306 (12.2)	2194 (87.8)	2500 (100)	na
Age in years †		76.9 (75.1, 78.6)	76.6 (76.0, 77.2)	76.6 (76.1, 77.2)	0.713
Gender	Male	136 (44.4)	1114 (50.8)	1250 (50.0)	0.038
AKI Stage	Stage 1	126 (41.2)	1227 (55.9)	1353 (54.1)	0.0001
	Stage 2	98 (32)	530 (24.2)	628 (25.1)	
	Stage 3	82 (26.8)	437 (19.9)	519 (20.8)	
Site of AKI	Community	284 (92.8)	1773 (80.8)	2057 (82.3)	0.0001
	Hospital	22 (7.2)	421 (19.2)	443 (17.7)	
Admission type	Elective	13 (4.2)	208 (9.5)	221 (8.8)	0.003
	Emergency	293 (95.8)	1986 (90.5)	2279 (91.2)	
Ethnicity	White	277 (91.1)	1994 (91.5)	2271(91.4)	0.915
	Mixed	2 (0.7)	6 (0.3)	8 (0.3)	
	Asian	8 (2.6)	60 (2.8)	68 (2.7)	
	Black	3 (1)	21 (1)	24 (1)	
	Other ethnic group	3 (1)	16 (0.7)	19 (0.8)	
	Not known	11 (3.6)	83 (3.8)	94 (3.8)	
Charlson’s score	0	95 (31)	625 (28.5)	720 (28.8)	0.468
	1	64 (20.9)	462 (21.1)	526 (21)	
	2	46 (15)	409 (18.6)	455 (18.2)	
	3	48 (15.7)	307 (14.0)	355 (14.2)	
	4	21 (6.9)	190 (8.7)	211 (8.4)	
	≥5	32 (10.5)	201 (9.2)	233 (9.3)	

^†^Mean (95% CI)

**Table 2:** Care bundle completion in stage 3 AKI and site of AKI.

	Care bundle within 24 hours. N (%)	Care bundle after 24 hours or not completed. N (%)	P value
Community acquired AKI	74 (90.2)	376 (86)	0.377
Hospital acquired AKI	8 (9.8)	61 (14)	

### Compliance with individual elements of AKI CB

Volume depletion was found in 67% of AKI patients, while detailed history was obtained in only 29.1% of patients. 52.9% of patients had their medications checked for nephrotoxins and 21.2% of patients were found to have nephrotoxic medications. Obstructive symptoms were present in 17.6% of patients, while sepsis was found in 36.3%. Urinalysis was performed in <12% of patients. AKI was classed as pre renal in 71.9%, renal in 9.8% and post renal in 4.5% of patients. Ultrasonography of kidneys was requested in 17.3% of AKI stage 2 or 3 or if urinary tract obstruction was suspected. In 70.9% of patients, appropriate treatment measures were instituted for example, stopping nephrotoxic medications, antibiotics for sepsis or relieving urinary tract obstruction. Monitoring fluid balance, blood tests and early warning sore was instituted in 70.9% of patients. Nephrology advice was obtained in 15.7% of patients and only 1.3% of doctors documented that they had referred to AKI guidelines on hospital website (S4 Fig)

Table 3 shows univariate analysis of outcomes between AKI patients grouped according to CB completion. The mean length of stay was lower as compared to that in patients who did not have CB completed, though this did not reach statistical significance, 11.2 (95% CI 9.9, 12.4) days and 12.5 (95% CI 11.9, 13.1) days, p = 0.1. AKI progression to higher stages was significantly lower in patients who had the AKI CB completed early. In patients with AKI who had the CB completed early, the in-hospital case-fatality was significantly lower at 18% versus 23.1% in those who did not have CB, p = 0.045. Though the 30-day and 60-day mortality was lower in patients who had the CB completed early, this did not reach statistical significance on univariate analysis. The case fatality after a median of 134 days was significantly lower in AKI patients who had CB completed early (30.1% and 36.5% respectively). During the same period the in-hospital mortality rate for patients without AKI was 1.24%. AKI progression to higher stages for patients with AKI stage 1 and 2 was significantly lower in patients who had the AKI CB completed early (3.9% versus 8.1%, p = 0.02).

**Table 3:** Univariate analysis of outcome with CB completion.

	Care Bundle completion		P value
	Within 24 hours	Not completed or completed after 24 hours	
Proportion of AKI episodes with progression to higher AKI stage	9 (3.9%)	149 (8.1%)	0.02
Length of stay in days†	11.2 (9.9, 12.4)	12.5 (11.9, 13.1)	0.098
In-hospital case fatality	55 (18%)	506 (23.1%)	0.046
30-day case fatality	77 (25.2%)	626 (28.5%)	0.219
60-day case fatality	83 (27.1%)	673 (30.7%)	0.205

^†^Mean (95% CI)

**Fig 2:**
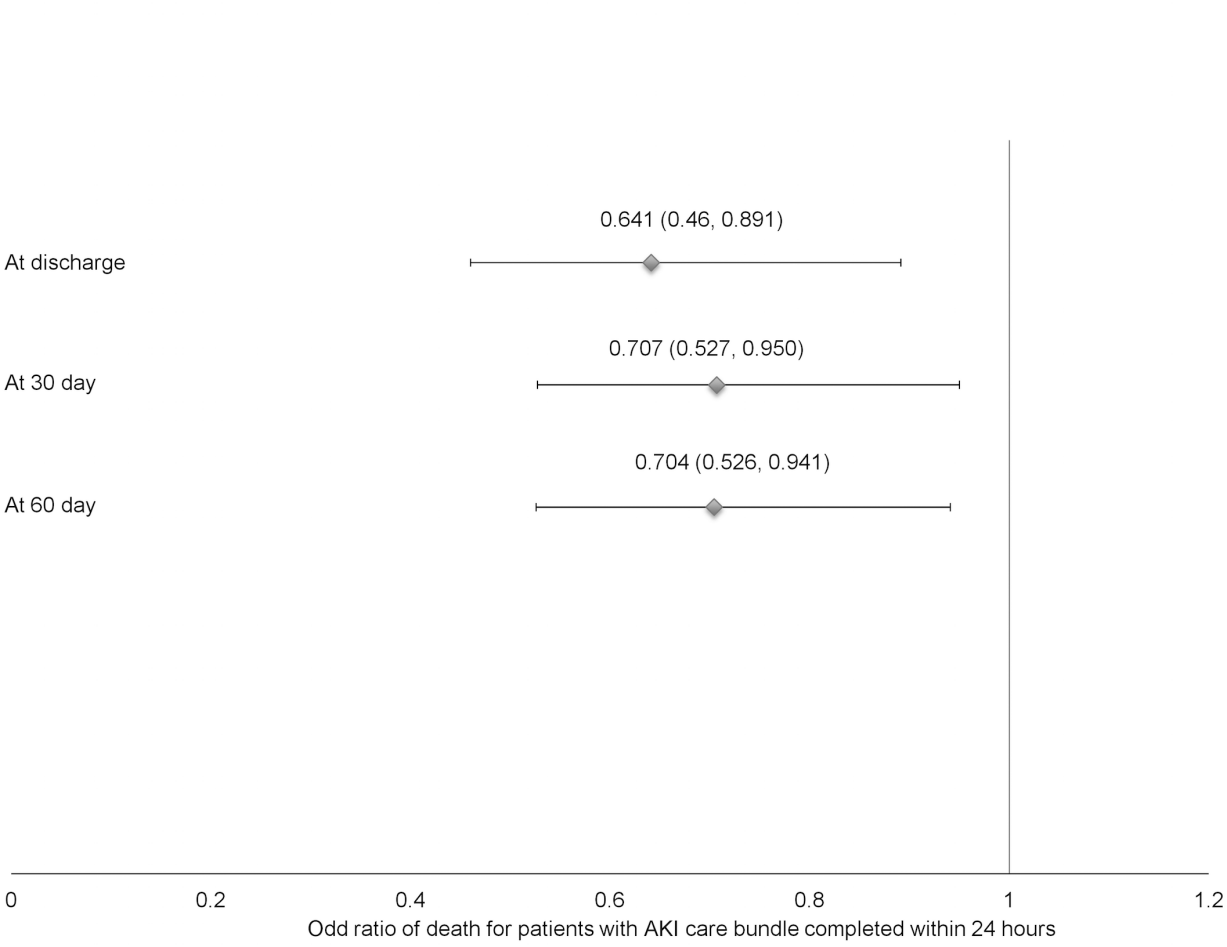
Odd ratios and 95% confidence interval of death for AKI episodes which had AKI Care Bundle completed within 24 hours.

**Fig 3:**
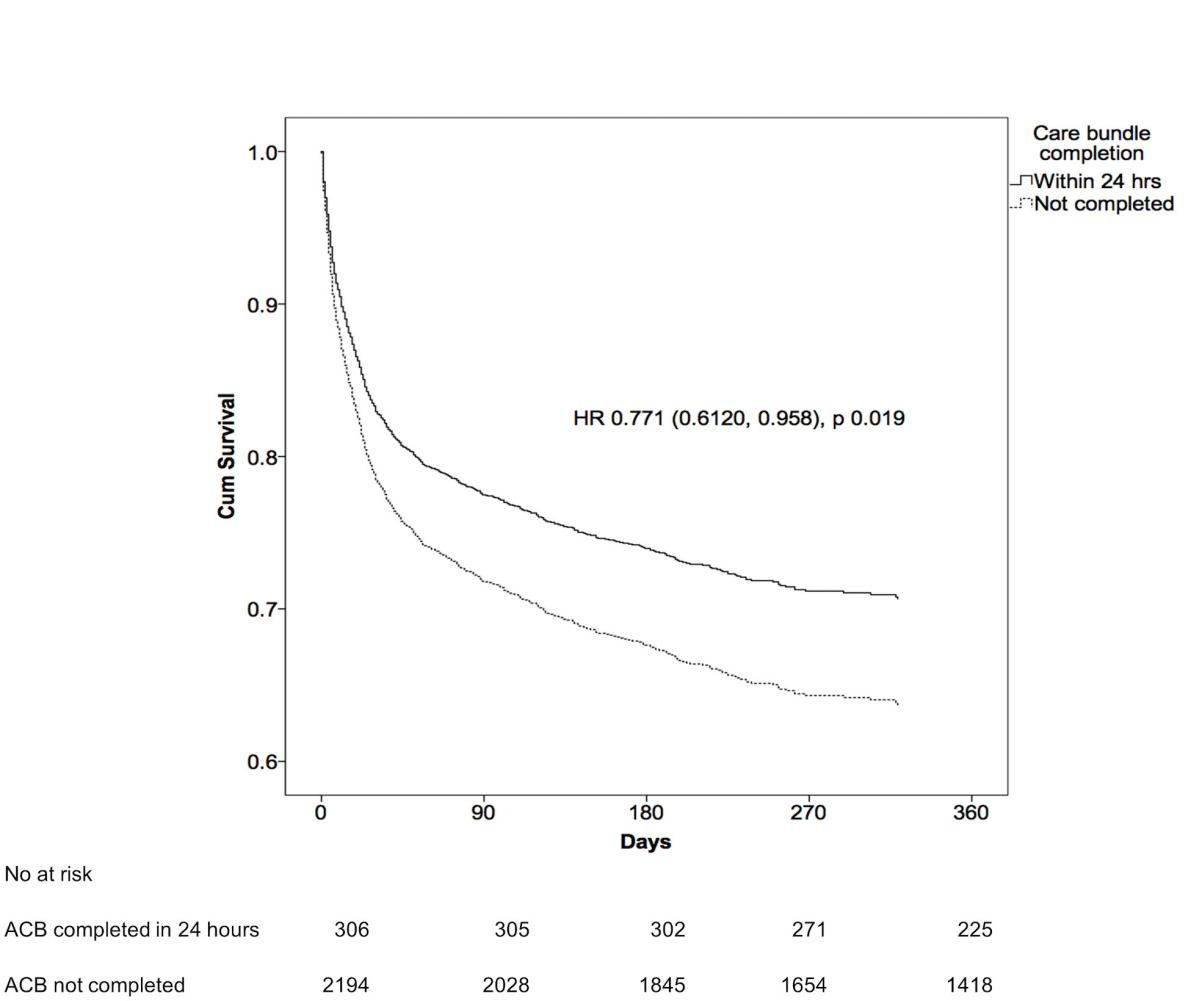
Adjusted survival curve stratified by timing of completion of AKI Care Bundle.

In the multivariate analysis, higher Charlson’s comorbidity score, incremental AKI stages, hospital acquired AKI and emergency admission were associated with higher odds of in-hospital death (Table 4). After adjusting for age, gender, type of admission, ethnicity and Charlson’s score, completion of the CB within 24 hours was associated with lower odds of in-hospital case fatality (OR 0.641; 95% CI 0.46 to 0.891), 30-day case fatality (OR 0.641; 95% CI 0.46 to 0.891) and 60-day case fatality (OR 0.641; 95% CI 0.46 to 0.891) (Fig 2). In Cox Proportional Hazards analysis, completion of CB within 24 hours of admission was associated with significantly lower hazard ratio of death after a median follow-up of 134 days of 0.771 (95% CI 0.620, 0.958) as compared to patients who did not have CB completed within 24 hours, p = 0.019 (Fig 3).

**Table 4:** Multivariate analysis of factors affecting mortality in patients’ with AKI.

Variables		Odd ratio (95% CI)	p value
Age		1.028 (1.019, 1.037)	0.0001
Sex	Male	Ref	
	Female	1.058 (0.863, 1.297)	0.588
Charlson’s score	0	Ref	
	1	1.817 (1.308, 2.523)	0.0001
	2	2.101 (1.515, 2.914)	0.0001
	3	2.729 (1.938, 3.842)	0.0001
	4	4.036 (2.766, 5.888)	0.0001
	≥5	4.36 (3.012, 6.312)	0.0001
Ethnicity	White	Ref	
	Mixed	0.286 (0.03, 2.68)	0.273
	Asian	0.552 (0.277, 1.097)	0.090
	Other ethnic group	1.451 (0.372, 5.651)	0.592
	Not known	0.69 (0.382, 1.248)	0.220
Max AKI Stage	Stage 1	Ref	
	Stage 2	2.354 (1.843, 3.007)	0.0001
	Stage 3	3.044 (2.355, 3.935)	0.0001
Admission type	Elective	Ref	
	Emergency	3.411 (2.01, 5.788)	0.0001
Place of AKI	Community AKI	Ref	
	Hospital AKI	2.056 (1.597, 2.647)	0.0001

## Discussion

In this analysis of observational data we observed more than 10-fold increase in the use of the CB for AKI, after introduction of an interruptive alert, which was linked to the electronic recognition of AKI. Furthermore, the completion of an AKI CB within 24 hours of abnormal blood test was associated with less progression to higher AKI stages and significantly lower in-hospital, 30-day and 60-day case fatality. The survival advantage was still present after median follow up of 134 days. Though not statistically significant, patients who had the CB completed early also had lower length of stay.

To improve recognition of AKI, there is a big drive to develop alerting systems, which can take the form of an electronic recognition on hospital laboratory systems or an active alert on handheld devices or mobile phones when a patient has AKI [10, 14–18]. The advantage of an active alert, directly to a responsible physician, is quicker relay of information especially if the alert is linked to real time staging of AKI [19]. However, there is a greater risk of false positive alert rate, alert override and provider non-adherence [20]. The presence of an electronic AKI alert makes a physician or a surgeon aware of the AKI event, but is not sufficient to initiate a therapeutic intervention, for example, volume replacement or stopping nephrotoxic medications [20]. This was evident in a study by Colpaert where the rate of therapeutic intervention fell significantly from 28.7% in alert phase to 10.4% in the post alert phase[18]. In our study, we found that introduction of the AKI CB alone was not sufficient as it was dependent on doctors looking for the AKI CB and completing it. The interruptive alert actively warned users about AKI from the electronic recognition system at the time of requesting any tests or ordering medications and prompted users to complete the CB. Such support has been recognized to improve outcomes in clinical practice [21].

The use of bundles of care interventions, as an approach to improving standardization and reliability of care received by patients with sepsis, has been demonstrated successfully for nearly ten years, with a growing body of published results in medical journals [22]. The first observational study of 6-h and 24-h sepsis bundles in patients with severe sepsis showed that non-compliance with sepsis care bundle was associated with 76% increase in the risk for hospital death[23].

Two publications have noted decreases in hospital mortality and length of stay associated with implementation of one or both Sepsis CBs[24, 25]. However, there has been no study using the same strategy in AKI for unselected admissions. Colpaert and colleagues developed a real time e-alert for AKI in an intensive care unit and enrolled 951 patients in three study phases: pre-alert control phase during which physicians were blinded to the electronic alert, alert phase, where physicians received an alert for every AKI episode and a post alert phase[26]. They found that higher proportions of patients received therapeutic intervention in the form fluids, inotropes or diuretics but no CB was introduced. They did not find any significant change in mortality or length of stay before and after the alert. A recent editorial has stressed the importance of developing clinical decision support systems to support electronic recognition of AKI and to improve outcomes [27].

We observed a significantly lower risk of in-hospital case-fatality in patients who had the AKI CB completed within 24 hours that persisted for up to a median of 134 days. This is important because AKI is associated with a high risk of death and no interventions have yet been proven to improve this [6, 8, 9]. We believe that the CB facilitated better basic care for a greater proportion of patients and that this resulted in improved outcomes. Unfortunately we were unable to test this hypothesis because we did not have data regarding the interventions actually applied in individual patients where AKI CB was not completed. Future studies should evaluate the impact of CBs on the care delivered and on outcomes. The interruptive alert reinforced the use of the CB and this was evident from the significant rise in the early use of CB from 2.2% to 21.6% after the alert. However, even in the presence of an interruptive alert, more than 75% of patients with AKI had no CB completed. Electronic recognition of AKI will detect AKI in databases, but will not alter important patient safety markers like length of stay and mortality, unless it is associated with improvement in basic care. Given the observed association between use of the AKI CB and lower mortality, it is important that we explore additional interventions including further education and feedback of AKI CB compliance rates to ensure that the CB is used for all patients with AKI. Making it more difficult or impossible to bypass the interruptive alert should also be considered.

### Strengths and Limitations

This is the first report demonstrating the effects of compliance with an AKI CB coupled with an interruptive alert in acute kidney injury in all hospitalized patients. Currently, AKI has no available therapeutics and this approach of improving outcomes in patients with AKI is important in all health care contexts, especially those without access to specialist or well developed medical care. There are several limitations to this study. This was not a randomized controlled study and the results may therefore been influenced by confounding factors. However, the group in whom the CB was completed early evidenced a higher proportion of AKI stage 3 and more emergency admissions suggesting that overall this group were at higher risk of adverse outcomes. This gives us greater confidence that our observation of better survival in the higher risk group who received the CB is robust. In the multivariable analyses we controlled for multiple potential confounders. This analysis of observational data therefore provides evidence that compliance with an AKI CB may lead to improved clinical outcomes but this requires confirmation with a propensity score matched study or a randomized controlled trial. Despite the use of an interruptive electronic alert the CB was completed in only a relatively small proportion of patients. However, this is unlikely to have affected the outcome because patients who had the the CB completed were similar in age, gender, ethnicity and comorbidity score to those without CB completion. In the cohort where AKI CB was not completed, we did not have information on appropriate assessment, diagnosis and timely treatment of sepsis, hypovolemia and so on. Patients included were predominantly elderly so further research would be required to confirm that similar benefits could be achieved in younger patients. On the other hand, the majority of patients with AKI are elderly and our data therefore are applicable to an unselected population of people with AKI. Finally this is a single center study, which may limit its external validity.

## Conclusions

We have observed that the use of an interruptive alert to improve compliance with an AKI CB to standardize and improve the initial management of AKI is associated with lower in-hospital, 30-day and 60-day case-fatality which persisted till a median follow up of 134 days. The AKI CB along with the interruptive alert can be implemented in any hospital with electronic results reporting, is simple to use and has the potential to improve patient outcome. A propensity score matched study or a randomised controlled trial is required to confirm these findings and future studies should focus on the impact of CB use on the quality of care delivered as well as outcomes. Methods should be developed to improve CB use further.

## Supporting Information

S1 Fig Paper AKI care bundle. (TIFF)

S2 Fig Electronic AKI care bundle. (TIFF)

S3 Fig Interruptive alert. (TIFF)

S4 Fig Compliance with AKI Care bundle. (TIFF)
