# Supplementary figures and images for: Impact of Compliance with a Care Bundle on Acute Kidney Injury Outcomes: A Prospective Observational Study
Source: PLoS One. 2015 Jul 10;10(7):e0132279. doi: 10.1371/journal.pone.0132279 (PMC4498890; doi:10.1371/journal.pone.0132279)

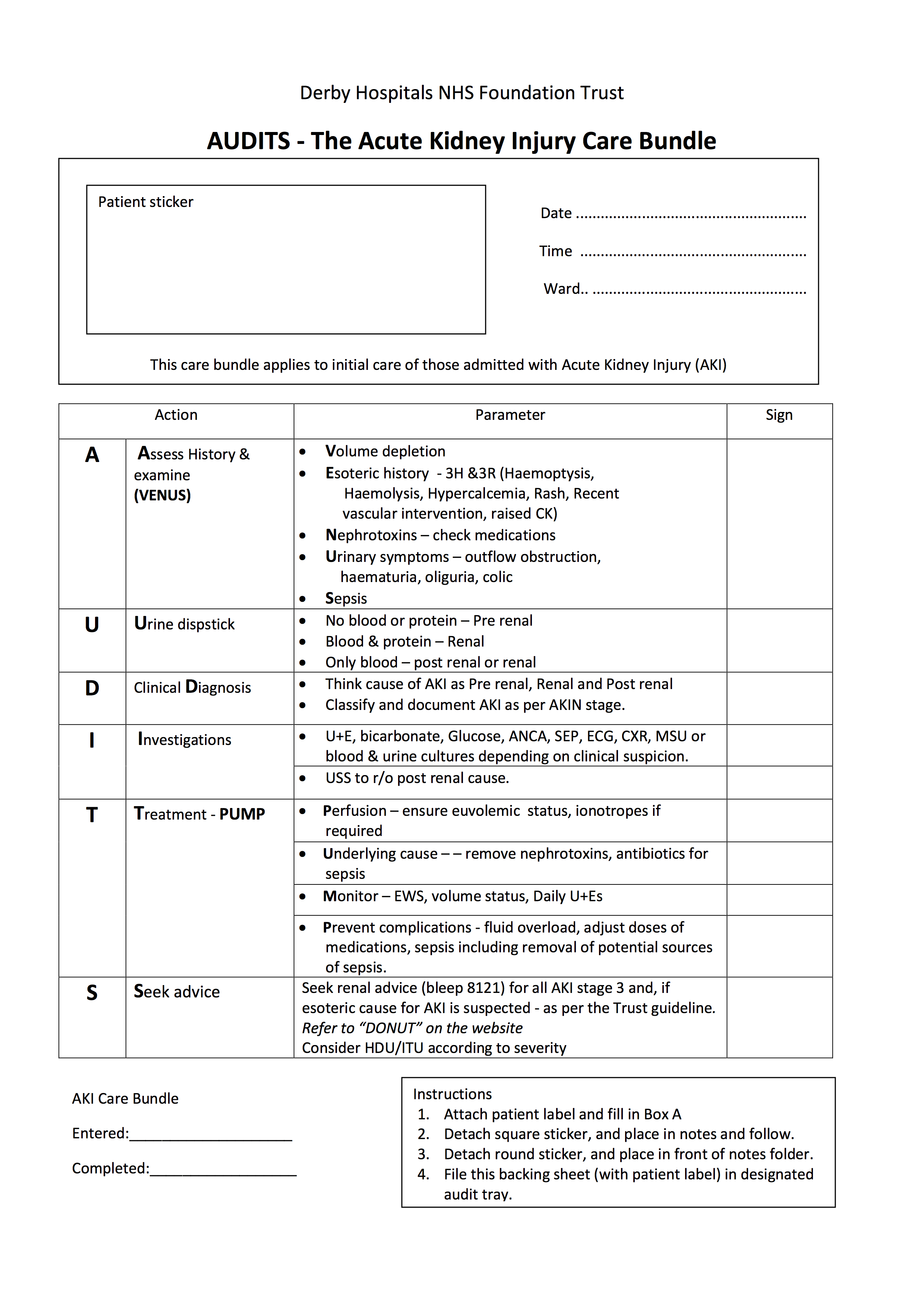

Supplement: S1 Fig — (TIFF) [file pone.0132279.s001.tiff]

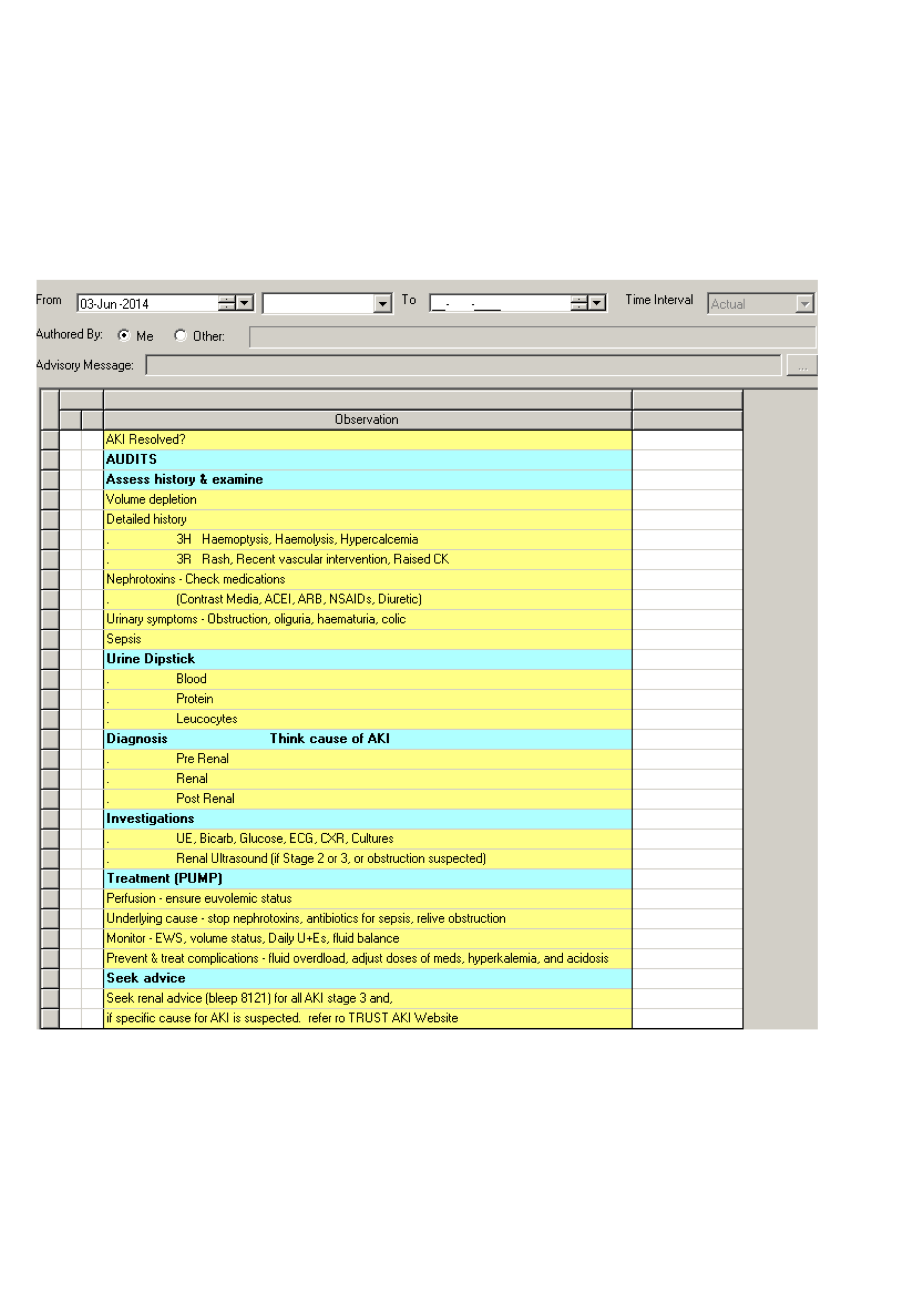

Supplement: S2 Fig — (TIFF) [file pone.0132279.s002.tiff]

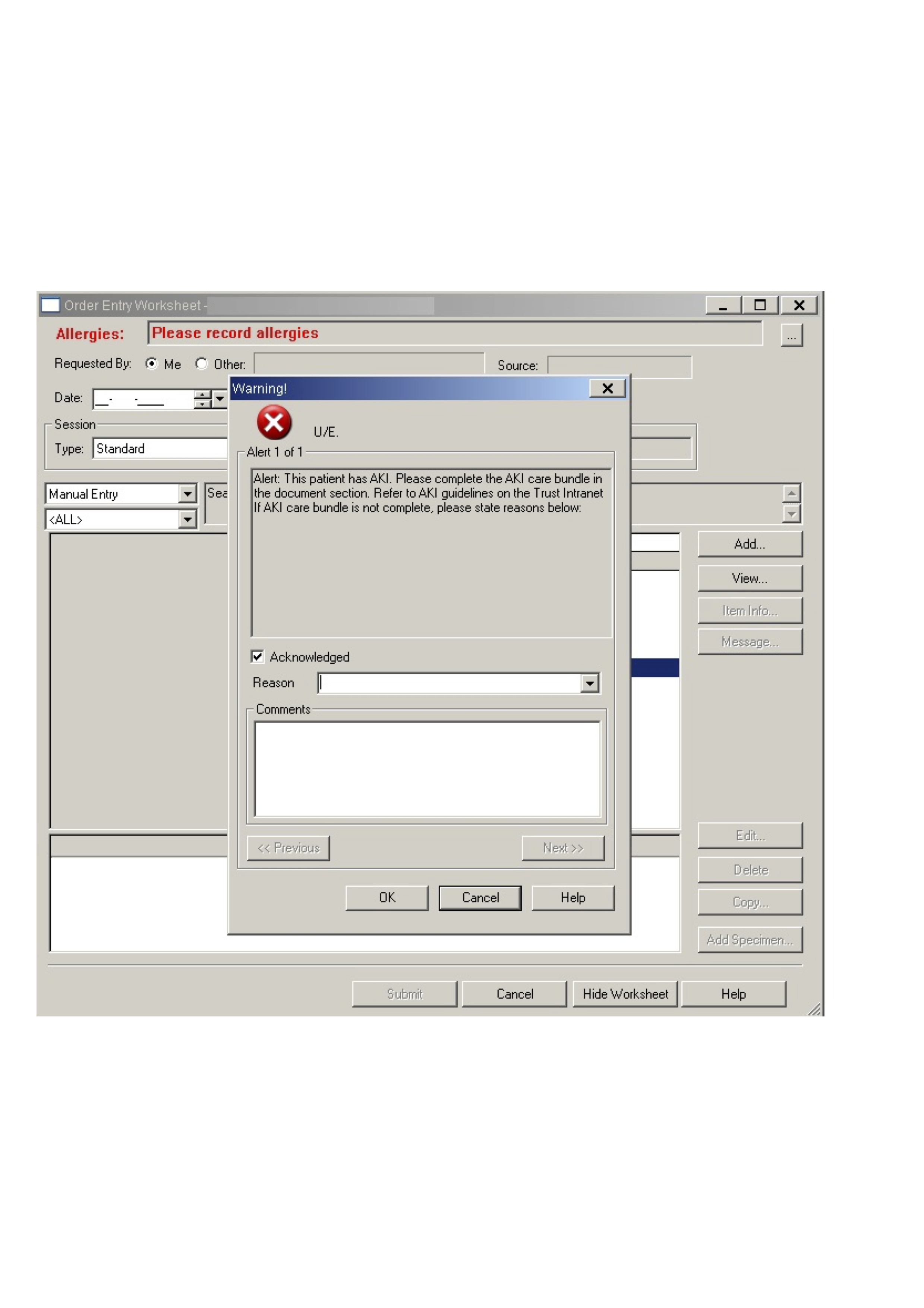

Supplement: S3 Fig — (TIFF) [file pone.0132279.s003.tiff]

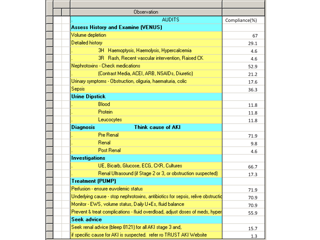

Supplement: S4 Fig — (TIFF) [file pone.0132279.s004.tiff]
